# Supplementary material for: Potential tradeoffs between effects of arbuscular mycorrhizal fungi inoculation, soil organic matter content and fertilizer application in raspberry production
Source: PLoS One. 2022 Jul 18;17(7):e0269751. doi: 10.1371/journal.pone.0269751 (PMC9292081; doi:10.1371/journal.pone.0269751)
Supplement: S1 Table — (PDF) [file pone.0269751.s001.pdf]

S1 Table. The number of replicated raspberry plants per treatment combination

Treatment combination			Number of plants produced fruits
AMF	SOM	Fertilizer (kg N·ha^{-1}·year^{-1})	
Inoculated	High	0	3
Non-inoculated	High	0	4
Inoculated	Low	0	5
Non-inoculated	Low	0	2
Inoculated	High	33	5
Non-inoculated	High	33	3
Inoculated	Low	33	4
Non-inoculated	Low	33	5
Inoculated	High	66	3
Non-inoculated	High	66	5
Inoculated	Low	66	3
Non-inoculated	Low	66	4
Inoculated	High	99	4
Non-inoculated	High	99	7
Inoculated	Low	99	5
Non-inoculated	Low	99	4
